# Peritonitis From Ruptured Lipid-Poor Dermoid: Struma Ovarii

**DOI:** 10.7759/cureus.16903

**Published:** 2021-08-05

**Authors:** Sai Swarupa Vulasala, Anastasia Singareddy, Dheeraj Gopireddy, Sindhu Kumar, Ketav Desai

**Affiliations:** 1 Radiology, University of Florida College of Medicine – Jacksonville, Jacksonville, USA; 2 Skin Biology and Dermatological Sciences, University of Miami Miller School of Medicine, Miami, USA; 3 Pathology, University of Florida College of Medicine – Jacksonville, Jacksonville, USA

**Keywords:** dermoid cyst, lipid poor dermoid, mature cystic teratoma, chemical peritonitis, struma ovarii, mri

## Abstract

Mature cystic teratoma (MCT) is a common benign ovarian germ cell tumor. It is more predominantly seen in premenopausal women and contains at least two or more well-differentiated germ cell layers. It is termed a dermoid cyst if the ectodermal tissue is the predominant component. The complications of a dermoid cyst include torsion, malignant degeneration, rupture, and infection. The incidence of a ruptured dermoid cyst is around 1%-2% resulting in chemical aseptic peritonitis from spillage of the cyst contents. Usual clinical presentation is with diffuse abdominal or pelvic pain and abdominal distension. Around 93-96% of dermoid cysts demonstrate fat in the cyst cavity however, minimal or no fat poses diagnostic challenges. In this case, we discuss a rare case of spontaneously ruptured lipid-poor and thyroid tissue-rich left ovarian dermoid presenting with chemical peritonitis. Special magnetic resonance (MR) Imaging sequences such as fat saturation imaging, chemical shift imaging, and gradient-echo imaging assist in detecting scant amounts of fat in the cyst cavity or cyst wall.

## Introduction

Teratomas are the most common ovarian germ cell tumors which arise from primitive germ cells [1]. Constituting 10%-25% of all ovarian tumors, they are frequently seen in premenopausal women around the age of 30-45 years [2]. Given the pluripotent nature of germ cells, teratomas demonstrate tissues from all three types of cell layers which include ectoderm (hair follicles, sebaceous glands, teeth), mesoderm (muscle, bone, cartilage, fat), and endoderm (thyroid tissue) [2]. Teratomas are most often seen in ovaries but they may also develop at other sites when germ cells become arrested during their migration from allantois to gonads [3,4]. Incidences of bilateral occurrence of teratomas range from 8% to 15% and they are unilocular in 90% of presentations [1,4]. The yearly growth rate is approximated to be 1.8 mm [5]. Asymptomatic and insidious growth of the tumor leads to delay in identification and the cases are often found incidentally upon imaging. Complications include torsion (16%), malignant degeneration (2%), rupture (1%-2%), and infection (1%) [6]. The spillage of contents from a ruptured cyst into the peritoneal cavity causes aseptic inflammation termed chemical peritonitis [1]. However, detection of chemical peritonitis may pose a diagnostic challenge especially if the cyst has a poor macroscopic fat content. In this case report, we present a 33-year-old female with sudden onset of abdominal pain diagnosed as chemical peritonitis secondary to spontaneous rupture of lipid-poor ovarian dermoid cyst.

## Case presentation

**Figure 1:**
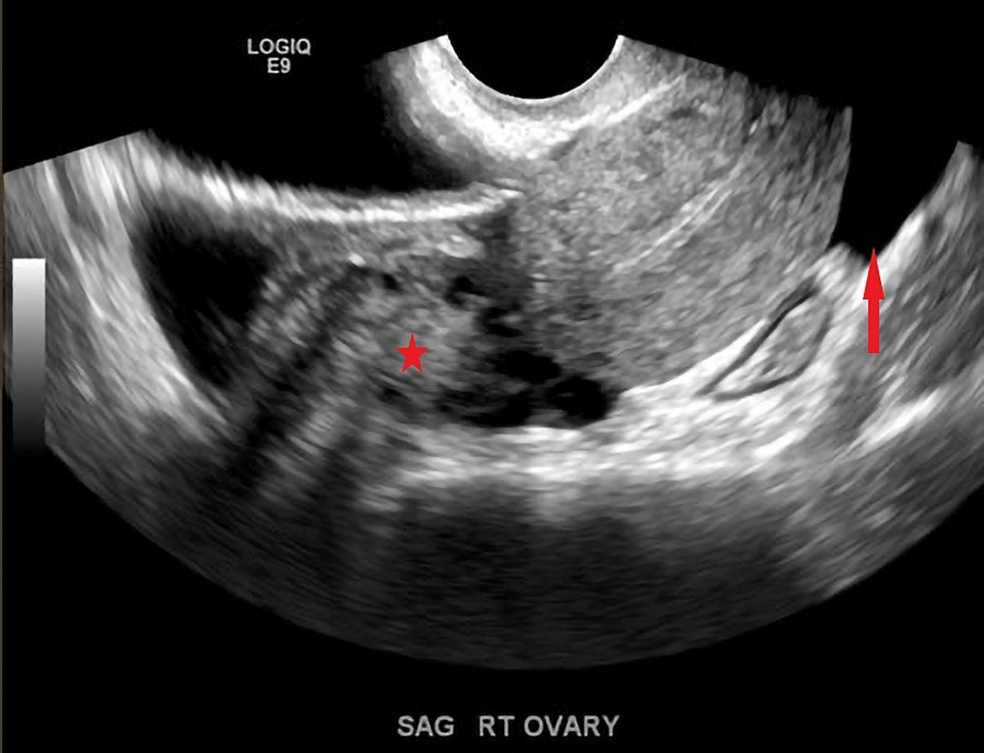
Transvaginal US grayscale images demonstrate a normal right ovary (star) and free fluid in the cul de sac (red arrow).

**Figure 2:**
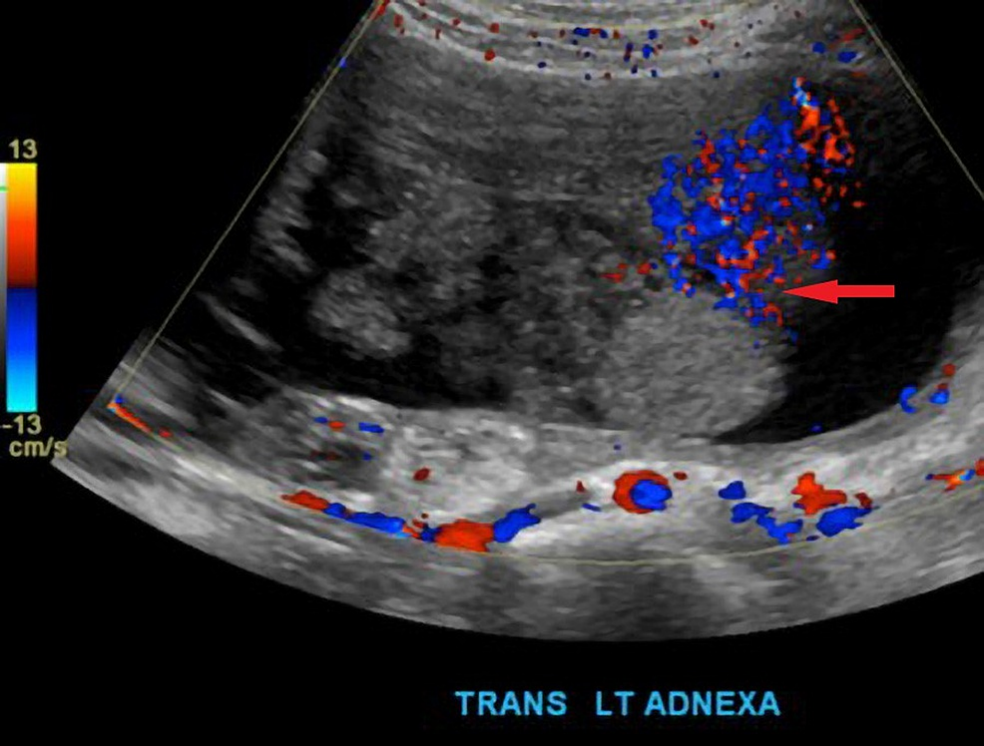
Magnification grey and color doppler images of the left adnexa show a complex mass with a cystic and solid component, dermoid plug (red arrow).

**Figure 3:**
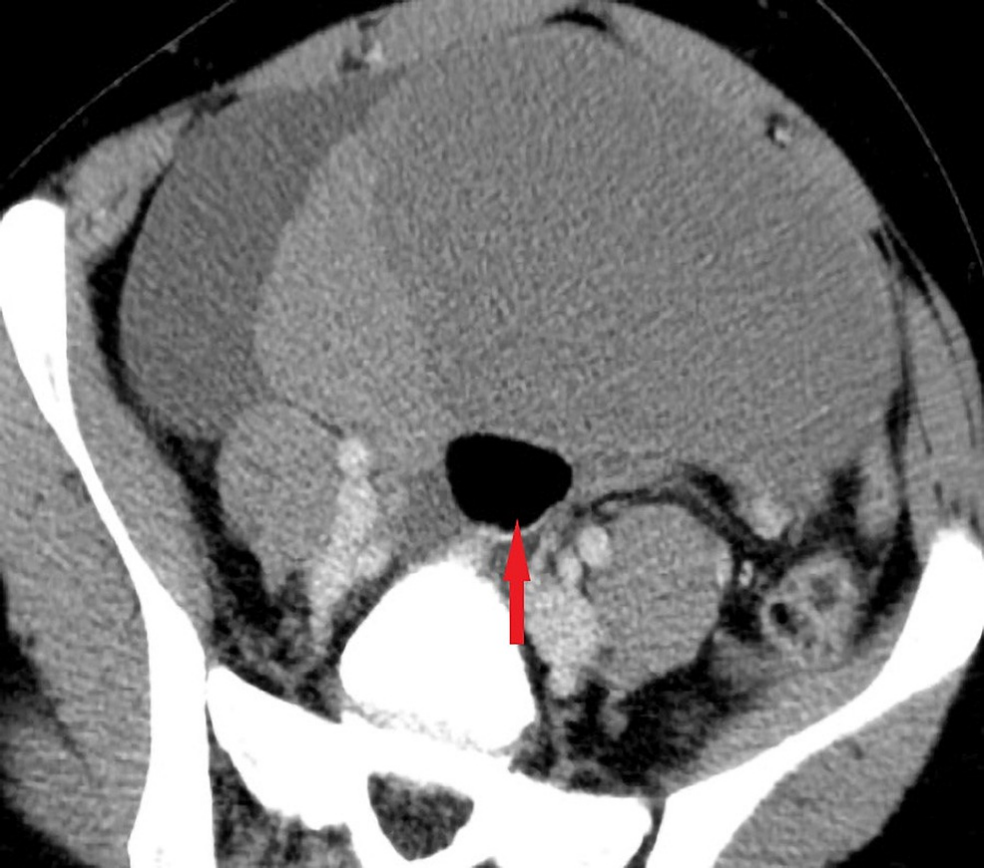
Axial post-contrast CT demonstrates a complex left ovarian mass with solid and cystic components, notice a globule of fat (red arrow).

**Figure 4:**
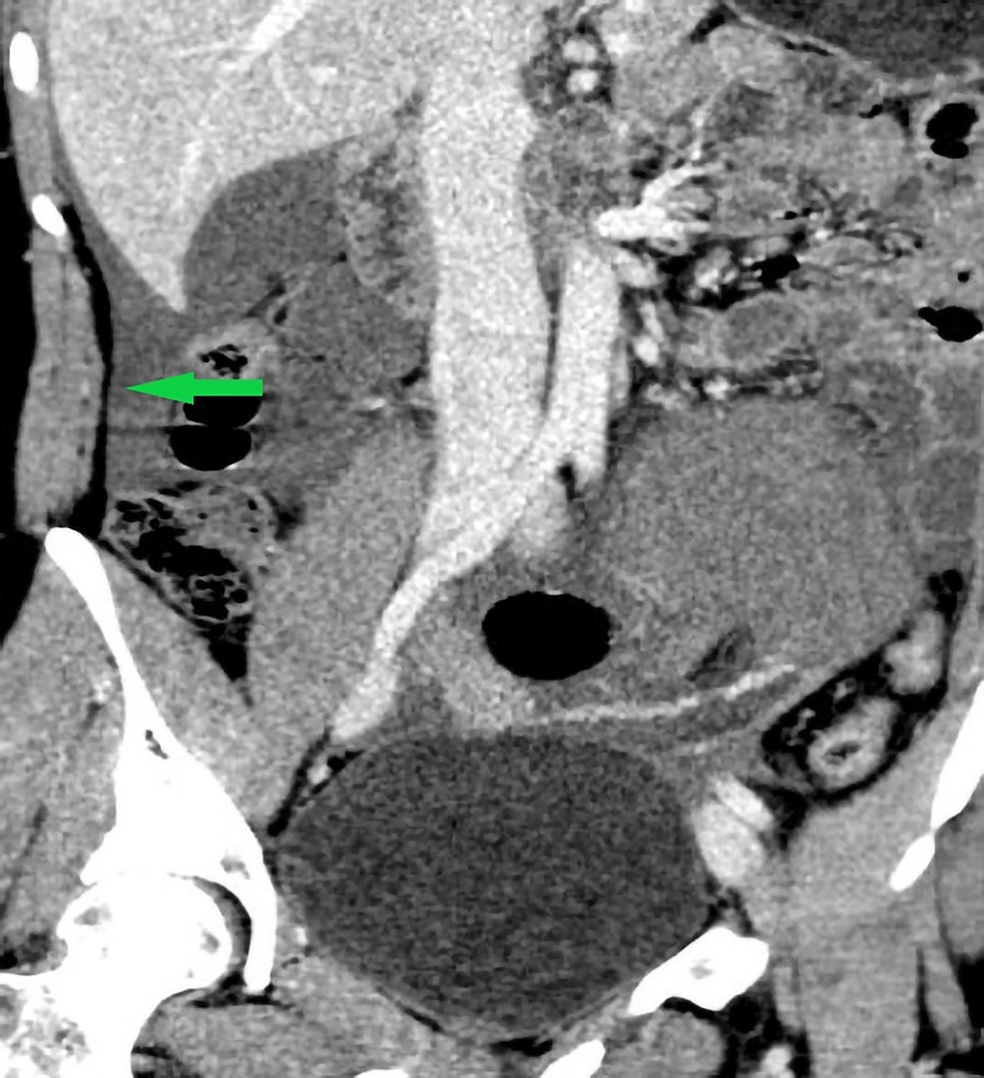
Coronal post-contrast CT demonstrates free fluid (green arrow).

**Figure 5:**
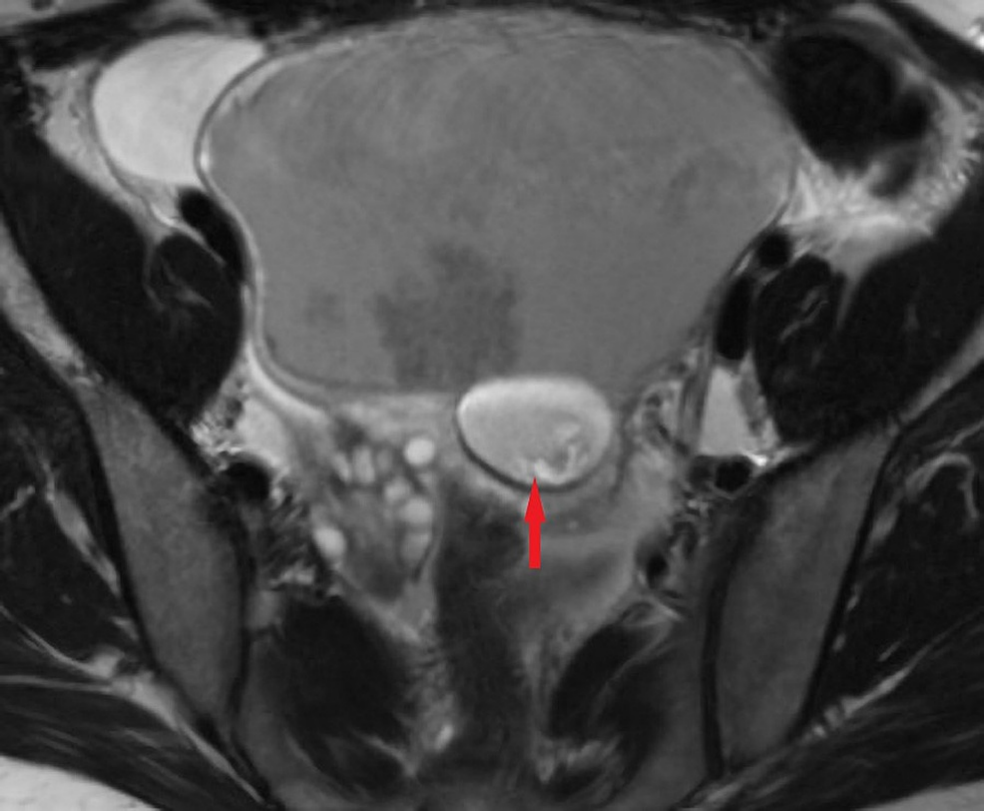
Axial T2 fast spin-echo without fat saturation demonstrates a complex cystic mass in the left adnexa with fat globule (red arrow).

**Figure 6:**
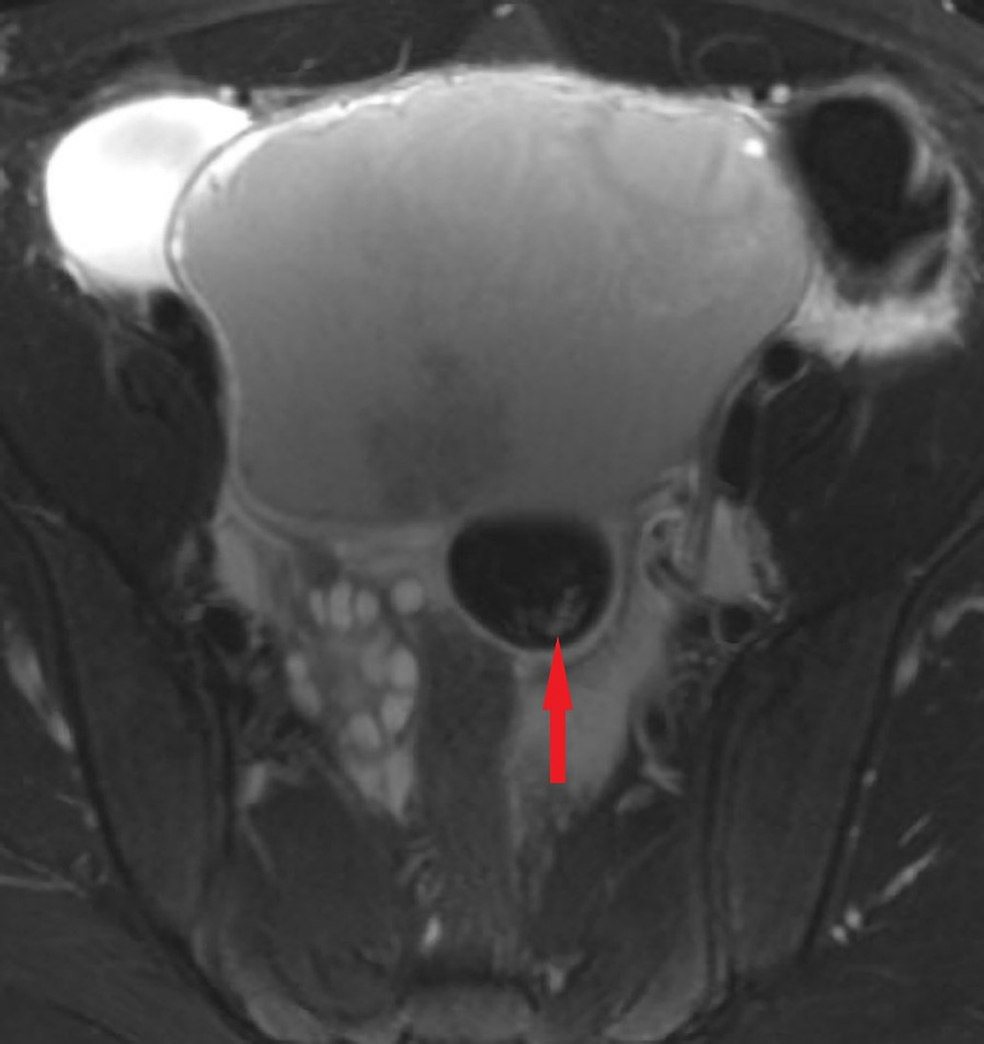
Axial T2 fast spin-echo with fat saturation demonstrates a complex cystic mass in the left adnexa with fat globule showing loss of signal on fat saturation images (red arrow).

**Figure 7:**
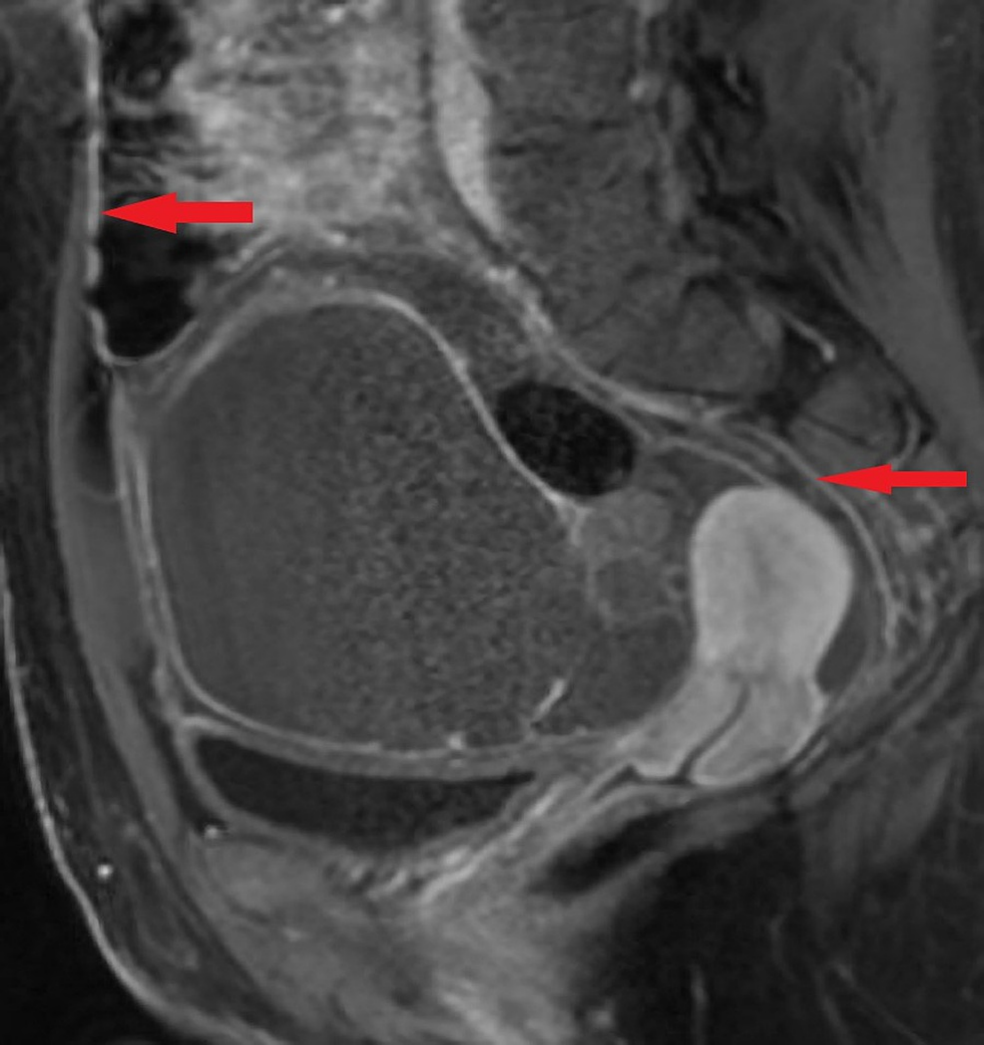
Post-contrast sagittal image demonstrates a complex cystic mass in the left adnexa with fat globule and smoothly enhancing peritoneum indicating chemical peritonitis (red arrows). No fat deposits were identified in the peritoneal cavity.

A 33-year-old nulligravida presented to the emergency department with acute onset of non-radiating left lower quadrant abdominal pain. The patient reported worsening pain with movement, which gets relieved by lying still. She also reported a progressive increase in abdominal girth over the past several weeks. Denied new vaginal bleeding, discharge, or usage of contraception. Diagnostic imaging with ultrasonography (US), computerized tomography (CT), and magnetic resonance imaging (MRI) were performed in the emergency room and are described in Figures 1-7. Initial pelvic ultrasound showed a large complex cystic and solid mass. MRI was further pursued to evaluate for possible ovarian torsion or a ruptured ovarian neoplasm.

**Figure 8:**
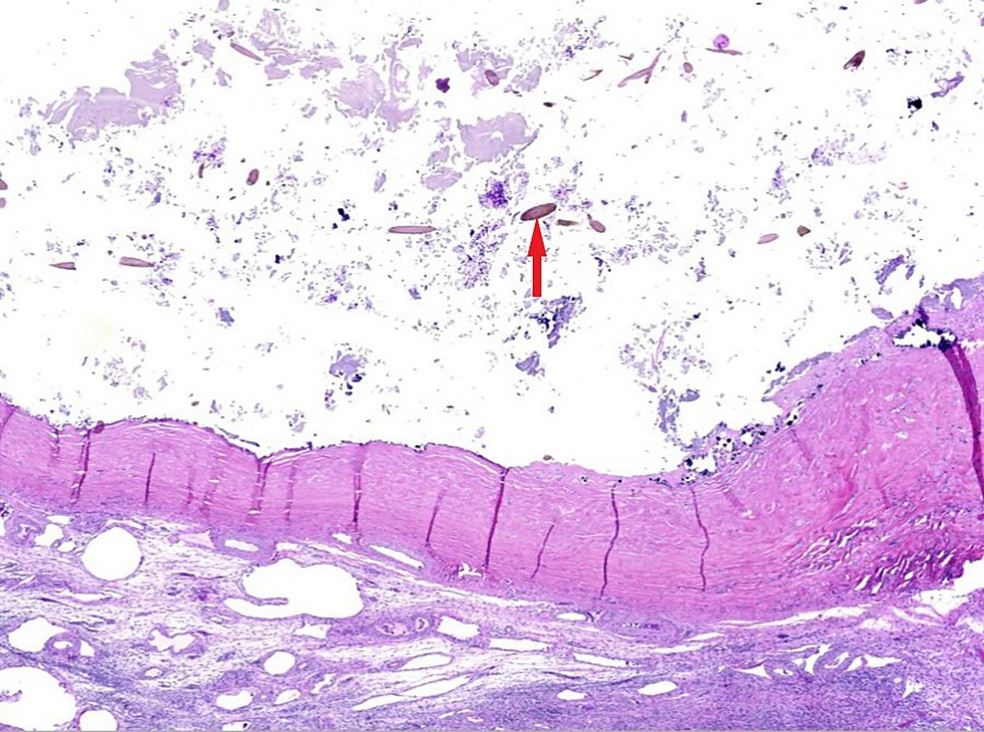
Cyst wall containing dystrophic calcification, keratin debris, and hair shafts (red arrow).

**Figure 9:**
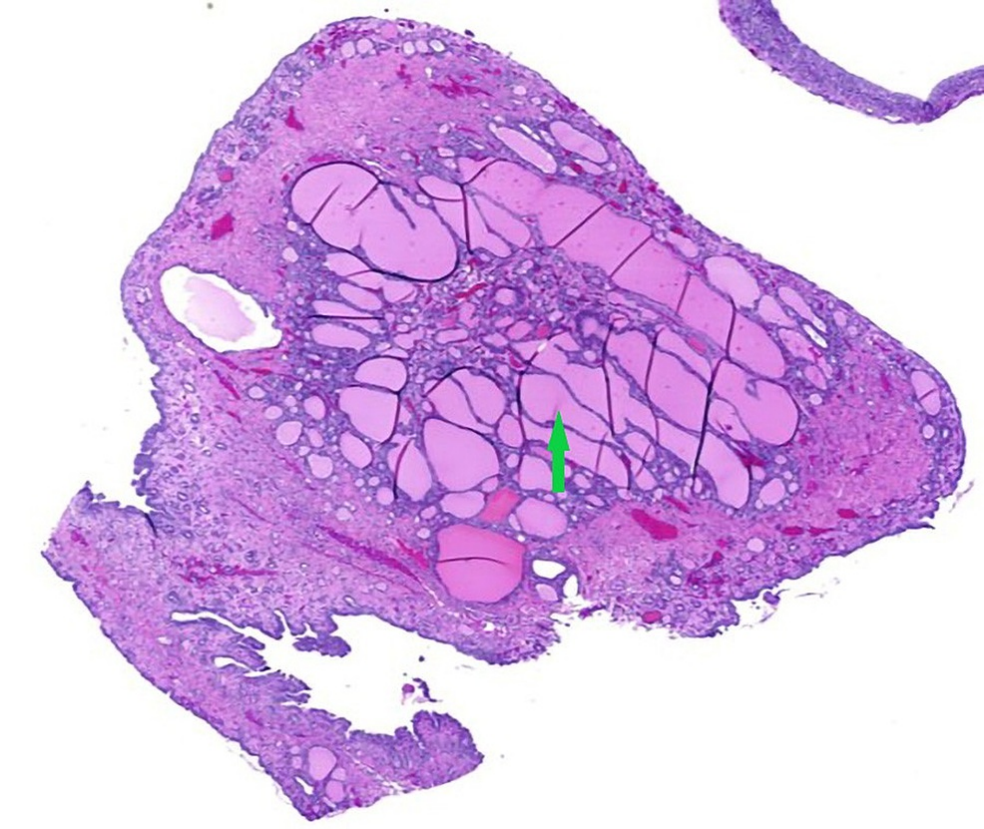
Benign thyroid tissue follicles of variable sizes, as well as abundant colloid material (Struma ovarii) lined with flattened epithelium (green arrow).

**Figure 10:**
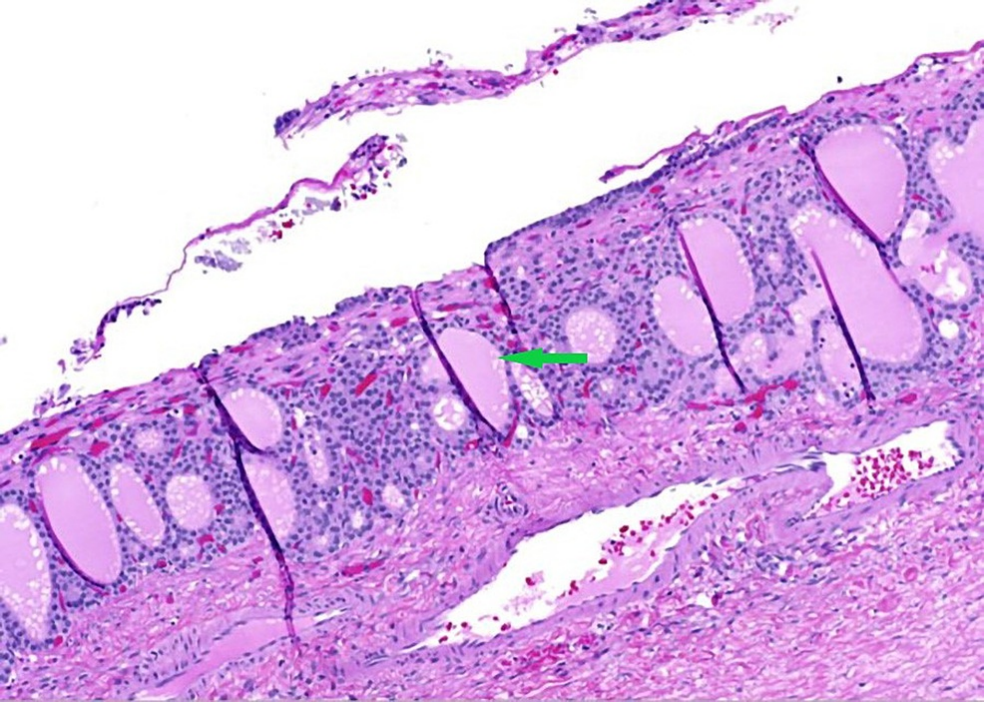
Benign thyroid tissue follicles of variable sizes, as well as abundant colloid material (Struma ovarii) lined with flattened epithelium (green arrow).

Discovering findings of a ruptured adnexal mass on imaging, the patient underwent exploratory laparotomy with left salpingo-oophorectomy. Intraoperative findings revealed a ruptured left ovarian cyst on the inferior aspect emanating serous fluid into the peritoneal cavity. The tumor was identified as mature lipid-poor cystic teratoma on the frozen section. The postoperative recovery was uneventful. The histopathology of the resected specimen is shown in Figures 8-10.

## Discussion

Ovarian germ cell tumors (OGCTs) are a group of tumors that include benign (mature cystic teratoma [MCT]) or malignant (immature teratoma, embryonal carcinoma, yolk sac tumor, dysgerminoma, and choriocarcinoma) neoplasms [3]. Teratomas are further classified as mature, immature, monodermal, and fetiform [5]. Of the teratomas, MCTs are the most common and are composed of at least two or more well-differentiated germ cell layers [5]. They are termed dermoid cysts if the ectodermal tissue predominates other germ cell layers [7]. Teratomas typically originate along the midline and Para-midline. Sacrococcygeal region involvement is seen in infants and children whereas the gonadal region predominates in adults [4]. The progression of a dermoid cyst to malignancy is rare, it is reported in 1%-3% of cases [8] and is more common in children and postmenopausal women. The dermoid cyst encloses sebaceous fluid secreted by the keratinized squamous epithelium of the thick cyst wall. The intratumoral fat (macroscopic fat or sebum) is the most characteristic finding of the dermoid cyst and is seen in around 93%-96% of the cases [9]. Hence a key image identifier on cross-sectional imaging especially CT. Other cyst contents include calcifications (56%), hair (65%), tooth, bone, and soft tissue [4,5]. Although rupture of a dermoid cyst is very rare, it may lead to spillage of its contents into the peritoneal cavity causing chemical peritonitis [1]. This rupture may occur abruptly or chronically due to minor tears in the cyst wall [10], our patient did not report trauma but presented with subacute symptoms over a few days. The manifestations of either acute or chronic include ascites and inflammatory nodules involving omentum and peritoneum mimicking peritoneal carcinomatosis or tuberculous peritonitis [11]. In the present case, there was a smooth enhancement of the peritoneum without nodularity as in Figure 7 which can be explained by more serous content of the dermoid cyst. Chemical peritonitis can lead to further complications such as fistula and adhesion formation requiring several surgeries [8]. Summary of key imaging findings of the dermoid cyst is described in Table 1 [5].

**Table 1:** Imaging findings of dermoid cyst. US: ultrasonography; CT: computerized tomography; MRI: magnetic resonance imaging; HU: Hounsfield units.

Imaging feature/sign	Incidence	US	CT	MRI
Rokitansky protuberance	81-86%	Hyperechoic with shadowing; Hyperechoic nodule protruding into the lumen	Rounded nodule protruding into the lumen/ as a mural thickening/cystic /tooth	Rounded nodule protruding into the lumen/as a mural thickening/ cystic
“Tip of the iceberg” sign	4%	Sebum, hair, cellular debris creating an echogenic focus that causes acoustic shadowing		
“Dot-dash sign”	61%	Hyperechoic dots or lines according to the orientation of hair along the imaging plane		
Fat-fluid level/fluid-fluid level	8-12%	It is described as either anechoic sebum floating on hyperechoic fluid or hyperechoic sebum floating on hypoechoic fluid	Hypodense fatty layer on the hyperdense dependent fluid layer	Hyperintense fatty layer on T1 and T2 weighted images with the signal drop on fat suppression imaging
Floating ball sign	rare	Floating hyperechoic globules that change their position with patient movement	Floating globules within a cyst in gravity independent position	Floating globules within the cyst in gravity-dependent position
Comet-tail appearance	12%	Hypoechoic hairballs with acoustic shadowing		
Intratumoral fat	93%	Hyperechoic	Hypodense with HU between -144 and -20	Hyperintense on T1 and T2 with the signal drop on fat saturation
Tooth/calcification	56%	Hyperechoic with posterior shadowing	Calcification is seen either in the Rokitansky nodule or cyst wall or in the septa.	A dark signal on T1 and T2
Chemical shift artefact	86%			High and low signal intensities on the opposite sides of the tumor
Tuft of hair	65%	Hyperechoic		As an artefact in the gravity-dependent portion of the cyst
Palm tree-like protrusion	21%			Mass resembling palm tree that is protruding into the cavity of the cyst
Intratumoral keratinoid material	75%			Hypointense on T1 and hyperintense on T2, Restricted diffusion on DWI

Ultrasonography (US) is the first line of imaging used in the evaluation of adnexal masses [12]. Differential diagnosis of cystic ovarian lesions includes follicular cyst, corpus luteum cyst, ovarian endometriomas, cystic teratoma, tubo-ovarian abscess, cystadenoma of the ovary, and cystadenocarcinoma. Sonographic features vary depending on the cyst contents as described in the Table 1. In our case, the US demonstrated a classic dermoid plug (Figure 2). The “dot-dash sign” (“dermoid mesh sign”) represents the alignment of hair along the imaging plane with a positive predictive value of 98% [5]. Dots represent echoes from hair perpendicular to the plane and dashes represent echoes from hair parallel to the plane [12]. The acoustic shadow may be present due to the echogenic mass containing sebaceous material blended with fat, hair, and cellular debris within the cyst. This shadow is represented as “the tip of an iceberg” sign as it disguises the area behind the mass [5]. The presence of a fat-fluid level in the cul-de-sac can be very helpful to identify a ruptured dermoid. However, the current case was lipid poor and hence no complexity was noticed in the peritoneal fluid.

In combination with findings from US, the CT assists in additional fat detection with a sensitivity of 93%-98% [1]. The Hounsfield units of fat range from -144 to -20 HU [13]. The fat attenuation with or without calcification within the cyst is diagnostic of MCT [2,9]. Calcification is best assessed by CT and seen as coarse or fine linear patterns [4]. A US correlate seen on the current CT imaging was a dermoid plug with a globule of fat (Figure 3). In a ruptured cyst, the CT demonstrates fat layered ascites, fatty nodules in the peritoneum and around the liver, and omental stranding may be seen due to granulomatous inflammation. Considering the lipid poor nature of the dermoid in our case, there was no evidence of fat-layered ascites.

T1-weighted and T2-weighted MRI in MCTs will show a hyper-intense signal representing sebaceous fluid. It can be differentiated from hemorrhagic cyst by fat suppression, chemical shift artifact, or gradient-echo imaging. The fat becomes hypointense on fat-saturated MR (Figure 5, 6), whereas hemorrhage remains hyperintense [4]. The fat-fluid interface is typical of MCTs. Rare cases may show no or scant fatty tissue within the cyst but may contain fat in the cyst wall that can be well spotted by chemical shift imaging with in-phase and out-of-phase techniques [10]. Although fat saturation imaging can demonstrate scant fat, the out-of-phase technique has higher sensitivity [14]. Diffusion-weighted imaging (DWI) can be affirmative in providing differential diagnosis by unveiling even a minuscule chunk of fat [15]. Post-contrast images are useful to further depict the extent of peritonitis and ascites fluid (Figure 7). Table 2 summarizes the literature review related to previously published cases of lipid-poor dermoid [9,14,16].

**Table 2:** Published reports of lipid-poor dermoid.

Article	No. of cases	Location of fat	Investigation
Yamashitha et al [14]	7 of 12 cases	Minimal fat in the cyst wall	Identified by histology; Confirmed retrospectively with gradient echo sequences.
	5 of 12 cases	No fat	
Simoes et al [9]	1 case	Minimal fat in the cyst wall	Identified on histology; No findings on imaging
AlGhamdi et al [16]	1 case	Minimal fat in the cyst wall	Identified on Gradient echo opposed phase sequence; Confirmed by histology

This case study emphasizes the importance of a multimodality approach to complex pelvic masses that present with pelvic pain. Advancement imaging modalities like Pelvic MRI can detect acute complications from these masses like torsion or rupture. Hence, aiding in more accurate diagnosis and pre-operative planning. Surgery is performed for definitive diagnosis and treatment. In the current case of the ruptured teratoma leading to peritonitis, the patient underwent exploratory laparotomy and salpingo-oophorectomy with peritoneal lavage. The ascitic fluid was serous without significant fat content. Histologically, the tissue demonstrated abundant colloidal material and minimal fat consistent with Struma ovarii. The tumor is termed struma ovarii when the thyroid tissue is predominant and comprises >50% of the tumor [17].

## Conclusions

In the emergency room setting initial work up for pelvic pain in female patients starts with a good quality ultrasound examination. Upon discovery of a complex mass further evaluation should be done with a pelvic MRI with IV contrast. Pelvic MRI can help identify immediate complications and determine the correct surgical approach. Identifying components of MCTs across multiple imaging modalities assist in differentiating adnexal masses. The wide range of presence of fat in the dermoid cyst makes it easily detectable on CT and MR imaging. Although some cases demonstrate low fat, using special techniques such as fat suppression MRI plays a vital role in early detection and emergency management. Once rupture and peritonitis are suspected, patients should be managed by emergent laparotomy (lower spillage rate than laparoscopy) followed by cystectomy.
